# The impact of measures to curb COVID-19 on patient attendance at 10 hospitals in Machakos County, Kenya

**DOI:** 10.7189/jogh.11.05016

**Published:** 2021-07-10

**Authors:** Tabither Gitau, Moses Kamita, Elizabeth Muli, Sharon Mweni, Rebeccah Waithanji, Faith Mutisya, Peter Kirira, Ancent Nzioka, Jonine Figueroa, Francis Makokha

**Affiliations:** 1 Mount Kenya University, Directorate of Research and Innovation, Thika, Kenya; 2 Technical University of Kenya, Department of Computer Science and Technology, Nairobi, Kenya; 3 Machakos County, Department of Health & Emergency Services, Machakos, Kenya; 4 The University of Edinburgh, Usher Institute, Centre for Global Health, Edinburgh, UK

## Abstract

**Background:**

The COVID-19 pandemic has resulted in both direct and indirect impacts on patients and population health. To better understand the impact of the measures put in place by the Kenyan government on health care provision, this project sought to document and quantify the impact of the restriction measures on patients' attendance in Machakos County.

**Methods:**

Hospital attendance at 10 public hospitals were obtained including Machakos Level 5 Hospital (the county referral facility) and one health facility from each of the 9 Sub-counties of Machakos County. Data on outpatient and inpatient attendance from November 2019 to May 2020 were obtained and compared with a similar calendar period from the previous year. Key informant interviews (KIIs), focused group discussions (FGDs) and in-depth interviews were conducted with the hospital management personnel (n = 46), as well as with the patients (n = 453) who missed scheduled clinic appointments at Machakos Level 5 Hospital to understand the reasons behind the drop in attendance numbers.

**Results:**

Overall, there was a decline in the number of patient attendances compared to the prior calendar period. Outpatient attendance reduced by 24.7% in April 2020 (n = 39 704) compared with April 2019 (n = 52 731). Inpatient attendance reduced by 13.7% in April 2020 (n = 3298) compared with April 2019 (n = 2845). Declines in patient attendance were observed in all hospitals that had inpatient services. A great decline in attendance was noted among larger hospitals that run specialty clinics, which were suspended mid-March 2020 when the first case of COVID-19 was announced. Some increase in attendance was noted in May when most clinics resumed operations. Most hospital management staff highlighted the closure of clinics as the main reason for reduced attendance while patients added that they also feared contracting COVID-19 at the hospital and the stigma they would face should they be quarantined.

**Conclusions:**

The findings from this study provide evidence that the COVID-19 pandemic outbreak and measures put in place by the government to curb its spread disrupted the provision of health services in Machakos County. Efforts to minimize adverse impacts of indirect impacts on access to health care and preventative services to counter increased morbidity and mortality require attention throughout the pandemic.

The Coronavirus disease of 2019 (COVID-19) was first reported in Wuhan, China in December 2019 but has now spread globally impacting negatively on human health, health systems, and overall well-being including economic activities and household incomes. The disease is caused by the SARs-Cov-2 which has been described as a descendant of the SARs-Cov-1 that also utilizes the human ACE-2 receptor to enter the host cell and similarly causes respiratory ailments [1].

Globally, 37 272 305 cases and 1 073 824 deaths of COVID-19 had been reported (https://coronavirus.jhu.edu/map.html) as of October 10, 2020. In Kenya, the first case of COVID-19 was reported on March 15, 2020, and as of October 10, 2020, 41 158 positive cases and 760 deaths had been reported by the Ministry of Health. Following reporting of the first case, the Kenyan government put in place several measures to curb the spread of the disease. These included public education on COVID-19 spread and symptoms, the importance of social distancing, closure of all academic institutions, 7 pm to 5 am curfew, travel restriction to and from selected counties (Nairobi, Mombasa, Kilifi, and Kwale), reduced the capacity of all public transport to half the original number, closure of restaurants and bars, and enforcing the compulsory wearing of masks in public space.

Changes in the transport sector resulted in increased costs for travel on most routes thereby increasing the financial burden of seeking health care. Additionally, imposed curfews and travel restrictions limited the ability to travel to referral hospitals located more than 60 km from patients’ residences. In developed countries such as the United Kingdom and USA, there is high adoption of digital health systems in hospitals and clinics enhancing storage and retrieval of medical information as well as patient follow-up [2]. However, such advancements are yet to be achieved in most of the developing countries where paper-based record keeping is still being practiced. Although Kenya has started the process of replacing paper-based health information management with digital ones, there is still more to be done to ensure real-time monitoring of patient trends [3,4]. This study aimed to document the trends of in-patients and out-patients attendance in 10 public hospitals in Machakos County and factors that influenced the continuity of health services within the county.

## METHODS

### Study setting

Machakos County borders Nairobi County and lies between the latitudes 0° 45’ South and 1° 31’ South and longitudes 36° 45’ East and 37° 45’ East. Machakos County has a total population of 1 421 898 people with a male to female ratio of almost 1:1, and 264 500 households [5]. The county covers an area of 6208 Square kilometres giving a population density of about 229 persons per square kilometre [5]. Machakos County was chosen for this study because it is one of the counties selected for the pilot Universal Health Coverage (UHC) programme in Kenya. The UHC package supports outpatient and inpatient services free of charge within the County. Also, the County government has heavily invested in the health sector including the purchase of 80 ambulances to strengthen the patients’ referral system. There are a total of 306 health facilities spread across the county and consist of 177 government, 103 private, 25 mission/faith-based, and 1 Non-Government Organizations.

### Study sites

**Figure 1:**
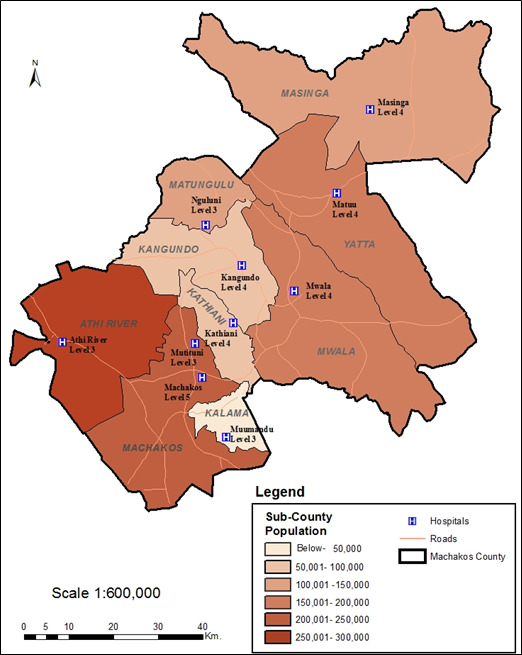
Map of Machakos County showing the nine sub-counties, the selected health facilities, and the catchment population and bed capacity for each health facility.

The study was conducted in all the nine (9) sub-counties of Machakos County, namely, Kangundo, Machakos, Athi-River, Yatta, Kathiani, Mwala, Matungulu, Masinga, and Kalama (**Figure 1**). In each sub-county, one health facility (**Table 1**) was selected based on capacity, catchment population, and accessibility.

**Table 1:** Selected health facilities at each sub-county in Machakos county, their catchment population and bed capacity

Sub-county	Health facility	Catchment population	Bed capacity
Kangundo	Kangundo	37,000	176
Machakos Town	Mutituni	10,985	4
Athi-River	Athi-River	38,000	11
Yatta	Matuu	36,913	42
Kathiani	Kathiani	16,814	129
Mwala	Mwala	17600	22
Matungulu	Nguluni	26,285	14
Masinga	Masinga	22,885	10
Kalama	Muumandu	7,620	13

### Patient attendance at the selected hospitals

Patients’ attendance data from the participating facilities were obtained through a mixed-method approach (qualitative and quantitative). The patients’ attendance data was obtained from the health records in the 10 selected health facilities. The health records officers (HROs) were sent data abstraction forms that they filled and submitted back to the research team. The numbers of inpatients and outpatients as well as those who attended each specialty clinic between November 2018 to May 2019 and November 2019 to May 2020 were recorded. Besides, data was collected on the availability of specialty clinics, hospital bed capacity, and catchment population. The data was then entered into the ODK Collect mobile application for transmission to the central database (Appendix S1 in the **Online Supplementary Document**).

### Qualitative data collection

The qualitative data collection was done to provide insight into a variety of issues related to service continuity before and during the COVID-19 pandemic, challenges, experiences, and opinions. In-depth interviews were done with patients to provide more information on their experience of specialty clinics before and during the pandemic, factors that hindered them from accessing specialized health services, their thoughts/views and availability on the possible referral to a hospital close to their residence. This was done through phone calls with the help of the nurses in the respective clinics to determine the reasons why the patients missed the clinic and identify the nearest health facility for referral purposes.

A total of nine focused group discussions (FGDs) were held one in each sub-county, with each group having 4-6 participants. The participants were purposively selected with the help of the medical superintendent in each facility ensuring that all the major cadres were represented. Each FGD comprised of a medical officer, clinical officer, health records officer, nurse, and pharmacist. They provided information on specialty health services in health facilities, factors that influenced service provision, measures put in place to ensure continuity of service provision, and possibility of referral of patients to the facility. A pilot study on the qualitative tool was conducted on 5 health care providers at Machakos Level 5 hospital (2 nurses and 3 doctors) after which a few additions and corrections were made to ensure that the questions were correctly interpreted. Key informant interviews and FGDs were conducted by the study team members using pre-set guiding questions (Appendix S1 in the **Online Supplementary Document**). Seven (7) Key Informant Interviews (KIIs) on consultants and nurses were held at Machakos Level 5 Hospital, the county’s referral hospital (**Table 2**). In-depth interviews (IDIs) (453) with patients were done on phone. The patients interviewed were those patients who were booked for clinic in either diabetic, hypertension, or cancer clinic in the month of March, April, and May, and did not attend the clinic. Diabetic clinic booking took place every Wednesday and Friday of the week while hypertension clinics took place every Monday, Wednesday, and Friday of the week. Cancer patients had monthly bookings although not on a specific day in the week. The interviews with the patients were conducted by health record officers working at Machakos Level 5 Hospital using a structured questionnaire as a guide. The health record officers were recommended to the study team by the staff in-charge of health records department and were engaged for two weeks to do patient follow-ups. The qualitative interviews were digitally recorded, transcribed (and, where applicable, at the same time, translated into English and independently checked by someone not involved in transcribing). Emerging themes were used to code the transcripts. NVivo software was used to support the analysis of the data.

**Table 2:** Overview of methods and the number of participants

Method	Participants	Number of participants in session	Total number of participants per data collection time
FGDs (max 4-6 participants per group)	Kagundo-5	39	39
	Mwala-5		
	Athi-River-4		
	Muumandu-2		
	Kathiani-5		
	Matuu-5		
	Masinga-5		
	Mutituni-4		
	Nguluni-4		
IDIs with patients		Cancer-13	748
		DM-212	
		HT-293	
		Paediatrics -230	
KIIs	Consultant:	7	7
	Paediatrics -1		
	DM-2		
	Mental health-2		
	Hypertension-1		
	Cancer-1		

FGDs – focused group discussions; IDI – in-depth interviews; KIIs – key informant interviews, DM – diabetes mellitus, HT – hypertension

### Data analysis

After extracting data from our online data capturing tool, retrospective data collected in November 2018-May 2019 and November 2019-May 2020 was analysed where frequencies and proportions were done to determine the trends using SPSS version 21 (SPSS Inc, Chicago, IL, USA). *t* test was done to test for significant differences between patient’s attendance between November 2018-May 2019 vs November 2019-May 2020.

For the qualitative data, in-depth interviews and FGDs were digitally recorded, transcribed and where applicable translated into English and independently checked by someone not involved in transcribing. Content analysis of the data was carried out using a comprehensive thematic matrix which facilitated identification of common patterns and trends arising from the narratives. Emerging themes were added to the matrix and the matrix was used to code the transcripts. We used NVivo software (QSR International, Melbourne, Australia) to support the analysis of the data. Narratives were written on main themes.

## RESULTS

**Figure 2:**
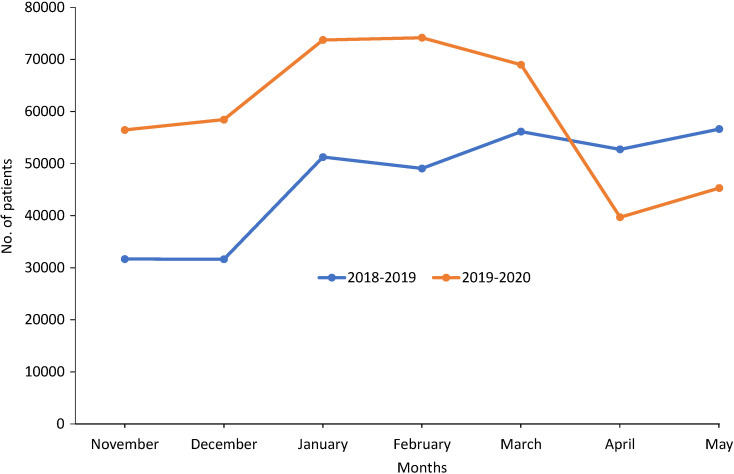
Trends of outpatients between November 2019 to May 2020 and the similar period previous year at all the 10 facilities combined.

Data on the overall outpatient attendance collated across all the 10 selected hospitals showed an increase in attendance in November 2019 to May 2020 period compared to the prior year until March 2020 when the first COVID-19 case was reported in Kenya (**Figure 2**). Outpatient attendance was higher in November 2019 (56459, 78% higher) than in November 2018 (31680) and then reduced progressively from January to April 2020. In April 2020, there was a decrease in the number of outpatients by 13027 patients (24.7%) compared to 52731 patients in April 2019. Inpatient’s attendance was generally stable from November 2019 to March 2020 followed by a decline in April 2020. This was a decline of 13% when compared to April 2019 in which 3298 inpatients attended. The overall outpatient’s attendance before the first COVID-19 case was announced in Kenya (November 2019 to February 2020) was significantly higher than similar period the previous year. These numbers reduced to no significant difference (Ρ<0.05) after the first COVID-19 case was announced (March 2020-May 2020) compared with similar period the previous year.

The number of outpatients in each of the selected facilities was lower in March and April before increasing in May 2020 (**Table 3**). In Machakos Level 5 hospital, for instance, there was a significant decrease in the outpatient attendance in April 2020 (44.3% decrease from March 2020) after which a slight increase was noted in May 2020 compared to the previous year where an increase was noted (**Table 3**). The decrease was likely a result of the cancellation of some of the clinics in March which saw fewer people attending clinics in March, April, and May 2020, and in some clinics no attendance was recorded in April and May 2020. The findings were supported by key informant interviews that indicated that indeed clinics were closed immediately the first case was reported.

**Table 3:** Outpatient attendance at selected health facilities in Machakos county between November and May 2018-2019 and 2019-2020*

Facility	Period	November	December	January	February	March	April	% change	May	% change
Machakos	2019-2020	25151	21675	28720	28136	25536	14225	-21.1	15443	-18.5
	2018-2019	10071	11230	16309	17005	18933	18028		18954	
Kathiani	2019-2020	6969	7556	6064	5910	6182	4788	19.9	5172	21.0
	2018-2019	2904	3204	3145	4184	4245	3994		4276	
Mwala	2019-2020	5872	6591	6995	7091	6703	4206	-38.3	4882	-23.2
	2018-2019	2713	3216	7474	4651	5308	6819		6356	
Mutituni	2019-2020	5234	5130	6622	6313	5841	3808	-6.0	5066	-7.2
	2018-2019	2327	2588	4126	3790	5021	4052		5459	
Kangundo	2019-2020	4301	4801	6761	6522	6117	3309	-39.8	3310	-34.1
	2018-2019	2622	3095	4496	5279	5589	5494		5019	
Matuu	2019-2020	694	3080	6024	6595	5808	3111	-36.4	3392	-36.6
	2018-2019	3165	2826	4135	4235	5586	4889		5347	
Nguluni	2019-2020	3263	3245	4786	4735	4592	1522	-52.3	3112	-26.9
	2018-2019	3279	1512	3943	3466	4021	3192		4260	
Athi River	2019-2020	1658	2824	3829	4358	3466	1889	-31.3	2080	-33.5
	2018-2019	2754	2210	3660	3294	4007	2748		3129	
Masinga	2019-2020	1595	1763	2515	2766	2874	1613	-20.0	1553	-27.9
	2018-2019	1060	1055	2619	1864	1879	2017		2154	
Muumandu	2019-2020	1722	1779	1421	1742	1855	1233	-17.7	1291	-22.8
	2018-2019	785	692	1352	1299	1549	1498		1673	

*% change indicates change in the number of patients in 2020 compared with the attendance in the same month in 2019.

“Changes have been there. Before COVID, the numbers were large but at the moment, the numbers have reduced. The way we have been continuing, the numbers have started to increase. Before COVID, the rough estimate was like 50 to 70. The clinic was held on Thursday. So, after COVID set in, it reached a point that we had to stop the clinic to prevent the exposure because the mentally ill patients cannot observe the social distance or wearing masks. As time went by, they accepted and coped with the situation... After putting all the measures-after one month- the clinic resumed. But still, the numbers were much lower than they were.” *(Nurse)*

“The number of patients that we had seen before and the number that we are seeing right now, there is a decrease, especially in some departments. In outpatients, of course, there are those departments that we had suspended. For example, the clinics, the MOPC (Medical Outpatient Clinic), POPC (Paediatric Out-Patient Clinic). But there are also some other departments that we got some influx of patients. Especially around the end of April and May, maternity was one of that.” *(Medical superintendent)*

In some clinics, there was a gradual reduction in the number of patients as more patients found it hard to access the health facilities due to the strict regulations imposed.

“…. It is only that before COVID-19, we used to have very big numbers of patients even ranging from 50 per day. There are times when patients come 40 of them…. The last clinic we had last week, we only had 13 patients. The previous week we had only 5 turned up.” *(Nurse)*

Similar trends were observed in all the other facilities as shown in **Table 3** except Kathiani hospital where an increase in patient attendance was recorded in both April and May 2020 compared with the same months in 2019. Changes in percentage for the months of April and May 2020 compared with attendance in the same months the previous year are indicated in **Table 3****.**

**Figure 3:**
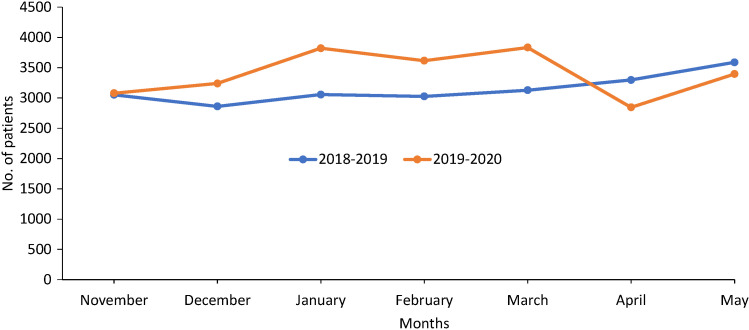
Trends of inpatients between November 2019 to May 2020 and the similar period previous year at all the 10 facilities combined.

The number of inpatients was also affected (**Figure 3**) although the effect differed from one hospital to another (**Table 4**). In Machakos, Matuu, Kangundo, Mwala, Athi River, and Mutituni hospitals, there was a reduction in the number of inpatients in April 2020 compared with the numbers in April 2020. On the other hand, Kathiani, Masinga and Nguluni hospitals recorded an increase in inpatient attendance in April 2020 compared with April 2019. This was probably occasioned by the restrictions imposed on accessing Machakos Level 5 hospital forcing many patients to seek health care at these lower-level facilities which are nearest to them although they do not offer some of the services needed by most chronically ill patients. Key informant interviews pointed out that there was also the possibility of patients being forced to seek health care at the nearest facilities due to restricted movements.

**Table 4:** Inpatient attendance at selected health facilities in Machakos county between November and May 2018-2019 and 2019-2020

Facility	Period	November	December	January	February	March	April	% change	May	% change
Machakos	2019-2020	1486	1330	1506	1446	1552	1240	-19.7	1251	-23.3
	2018-2019	1387	1394	1398	1465	1624	1544		1630	
Matuu	2019-2020	718	905	1182	1064	1214	704	-2.9	1163	38.5
	2018-2019	807	651	700	593	488	725		840	
Kangundo	2019-2020	495	534	610	593	552	385	-38.2	424	-28.1
	2018-2019	562	507	596	591	595	623		590	
Kathiani	2019-2020	104	187	161	178	151	200	53.8	221	19.5
	2018-2019	81	90	114	139	166	130		185	
Mwala	2019-2020	71	122	145	125	148	89	-3.3	161	21.1
	2018-2019	83	95	103	90	88	92		133	
Athi River	2019-2020	55	25	53	55	54	53	-23.2	58	-3.3
	2018-2019	57	54	82	50	62	69		60	
Masinga	2019-2020	60	54	63	80	62	60	27.7	52	-7.1
	2018-2019	55	41	50	61	58	47		56	
Nguluni	2019-2020	81	79	89	74	100	112	-86.7	58	-31.0
	2018-2019	11	20	13	35	45	60		84	
Mutituni	2019-2020	9	4	12	1	0	2	-75.0	8	-27.3
	2018-2019	10	9	1	2	3	8		11	
Muumandu	2019-2020	0	0	0	0	0	0	0.0	0	0.0
	2018-2019	0	0	0	0	0	0		0	

*% change indicates change in the number of patients in 2020 compared with the attendance in the same month in 2019.

“We have had increased deliveries. Both C-section and SVDs (spontaneous vaginal deliveries). We want to believe most of it, especially the introduction of roadblocks made sure that patients who used to go to the clinic in Thika and the rest of the larger metropolitan, actually coming to use to deliver and even the other patients in inpatients, that is what we have postulated was happening.” *(Clinical officer)*

In May 2020, Machakos, Kangundo, Athi River, Masinga and Mutituni recorded a decrease while Mwala, Kathiani and Nguluni hospitals recorded an increase in number inpatients compared with the numbers in May 2019 (**Table 4**). The quantitative findings are in line with our qualitative findings that documented a decrease in patient flow pre-Covid and during the pandemic. At health facilities level we looked at how COVID-19 has influenced service provision and it is a ripple effect. These observations were also supported by the Key Informant Interviews conducted at different health facilities. For instance, a participant at a Level 4 health facility noted that:

“Before this period, we had patients freely streaming into the hospital to the various departments where they wanted to get the services, and then after that, they would be discharged home or admitted or referred depending on the nature of their case. However, during COVID season patients visit to the hospital was only encouraged when you have something urgent this included emergency cases, accidents, and of course maternity services. At that time, we also stopped our outpatient clinics so that meant patients with chronic communicable disease had to be stopped from coming to the facility, there was that fear of, if they come, they may be exposed, and we know that those patients with comorbidities had a higher risk. Then the flow to the ward admissions has remained the same both pre and post Covid because you find that the condition that would warrant admission; there are conditions that would warrant one to be brought to the hospital and admitted. So that has not changed a lot.” *(Level 4 Health Facility)*

## DISCUSSION

From the results obtained, overall, there was a general increase in the number of outpatients from November 2019 to February 2020, compared to the same period the previous calendar year. This could be attributable to population growth dynamics as well as the adoption of Universal Health Coverage within Machakos County which was launched in December 2018 [6]. From March 2020, however, a steep decline was observed in the trend of patients seeking outpatient and inpatient services from all health facilities within Machakos County as seen in the results. This coincided with the period during which measures were instituted by the National Government and County Government to curb the spread of COVID-19 in Kenya. The measures included a Presidential directive of dusk (7 pm) to dawn (5 am) curfew announced on 27th March 2020 amongst others. The County Government of Machakos followed this directive a day later with the suspension of all outpatient clinics and elective procedures. On 6th April, the National Government ordered cessation of movement in and out of Nairobi to control the increase in cases numbers of COVID-19 [7]. This directive further hindered access to medical facilities such as Machakos Level 5 Hospital for patients residing in the areas demarcated as part of the larger Nairobi Metropolis. This influenced the continuity of health services which had a ripple effect on patients ie, reduction of patients accessing health services, reduction in uptake of services, increase in maternal deaths, fear, and deteriorating health conditions as reported elsewhere [8]. There was a bigger impact on the outpatient services as compared to inpatient services since inpatient facilities remained operational because patients requiring admission are mostly critical cases. Similar observations were recorded in Greece and England where a falling trend of the number of patients attending the emergency department was noted after restrictive measures were implemented to control the spread of COVID-19 [9-11]. The decline of inpatient and outpatients in April 2020 went below the numbers seen in the same month in 2019. This influenced the continuity of health services which had a ripple effect on patients ie, reduction of patients accessing health services, reduction in uptake of services, fear, and deteriorating health conditions. In some clinics, there was a gradual reduction in the number of patients as more patients found it hard to access the health facilities due to the strict regulations imposed. It is important to note that the observed changes in patient attendance took place even before the first COVID-19 case was reported in Machakos County (first case was reported on 9th May 2020).

The gradual increase in the number of inpatient and outpatient services observed in May could be as a result of the following factors: decline in initial fear of patients to access medical facilities, the opening of specialty clinics, reduction of curfew time, and measures like patient follow-up that were instituted by some health facilities. Some clinics reported an increase in patient attendance in May following the second directive by the Ministry of Health that relaxed the safety measures to reduce transmission of COVID-19 and reopening the outpatient clinics.

Facilities such as Machakos Level 5, Mutituni Level 3, Matuu Level 4, Athi River Level 3, Mwala Level 4, and Kangundo Level 4 hospitals showed a reduction in the number of outpatients both in the March and April 2020. Most of these hospitals have proximity to urban towns and major roads and were some of the hospitals where their catchment populations were affected by the government directives. The movement restriction and curfew law impacted heavily on these patients by limiting access to health facilities. Additionally, change in transport policy that was accompanied by an increase in the costs of public transport may have limited patients' ability to travel. On the other hand, facilities such as Kathiani Level 4, Nguluni Level 3, Masinga Level 4, and Muumandu Level 3 showed a reduction in the number of outpatients in April only. There is a possibility that most of the patients attending these health facilities were from communities residing around the hospitals and therefore were least affected by the cessation of movement and curfew directives.

In the case of inpatient services, some facilities like Kathiani Level 4, Mutituni Level 3, and Athi River Level 3 hospitals did not show any decline in the number of inpatients admitted. In some of these facilities, it was indicated that some departments experienced both an influx of patient flow and a decrease in some. An increase in maternal and child health services uptake was reported in some of the health facilities due to the lockdown restrictions where some patients on transit utilized immunization services and anti-natal clinic services.

Matuu Level 4, Mwala Level 4, and Machakos Level 5 Hospitals experienced a reduction in the number of inpatients in April 2020 only after which the numbers increased. It was only in Kangundo where the number of inpatients reduced both in March and April before raising again in May 2020. As stated earlier, Kathiani hospital is located within the community and therefore cessation of movement and curfew directives meant that patients who may have been seeking health services from other hospitals may have resulted in using the facility instead. Conversely, Matuu hospital which is in the Thika-Garissa road was receiving patients from different counties and therefore was more affected by the cessation of movement.

### CONCLUSION

The current study aimed to determine the impact of the measures that were set by the government to combat the spread of COVID-19 on the provision of health services in Machakos County. A total of 10 health facilities which included Machakos Level 5 hospital and one health facility in each Sub-county in Machakos county were involved. From the patient attendance trends, the data indicated a huge impact on the number of patients who registered as inpatient or outpatient at different facilities in Machakos county. April 2020 carried the heaviest burden of the impact where all the health facilities studied showed a reduction in the number of inpatients recorded and outpatient numbers in some of the health facilities. However, the situation slowly became better in May 2020 after the clinics were reopened. The lockdown measures imposed, and suspension of clinics were some of the major reasons that were cited by the hospital personnel interviewed in the FGDs. Although the measures taken by the government to combat COVID-19 spread bore fruits in reducing the number of people infected by the virus and its fast spreading to other parts of the country, the negative ripple effect was felt by those with other health conditions especially chronically ill patients whose clinics were suspended. These results highlight the need to keep the number of COVID-19 cases low in order to minimize both direct and indirect impacts on health. Unlike high income countries like United Kingdom or United States of America, hospital admission data tracking is still in its infancy in places like Machakos, a rural suburb outside Nairobi. In many countries (Sottish data) hospital admissions have been a key metric for determining the state of health care facilities and pressures due to the COVID-19 pandemic both for direct and indirect impacts. Here, we show using a quick data collection tool how such data could be collected and inform medical centers in places like Machakos. It is hoped that this will become a more routine practice throughout the pandemic but also beyond to monitor health services and assist in preparing for the needs of the population.

## RECOMMENDATIONS

- Closing/suspension of clinics should be discouraged to ensure the patients who require close monitoring continue receiving health care.
- Health facilities should have a patient follow-up system to ensure patients are adhering to their scheduled clinics and medication.
- To ensure the trends of patients attending the health facilities requires constant monitoring to ensure changes are detected early enough to give room for mitigation measures before it is too late.

## Additional material


Online Supplementary Document
