# Supplementary material for: The impact of measures to curb COVID-19 on patient attendance at 10 hospitals in Machakos County, Kenya
Source: J Glob Health. 2021 Jul 10;11:05016. doi: 10.7189/jogh.11.05016 (PMC8580289; doi:10.7189/jogh.11.05016)
Supplement: Online Supplementary Document [file jogh-11-05016-s001.pdf]

Supplemental Information

Appendix S1: Machakos County ODK Data Collection Tool

A. FACILITIES SUMMARY AND THEIR CAPACITIES

1. Select Facility name (choose only one)

   ○ Machakos Level 5

   ○ Kangundo Level 4

   ○ Kathiani Level 4

   ○ Matuu Level 4

   ○ Mwala Level 4

   ○ Masing Level 4

   ○ Mutituni Level 3

   ○ Athi River Level 3

   ○ Muumandu Level 3

   ○ Nguluni Level 3

   ○ Other (Specify) ___________________________________

2. Record the facility's location (GPS coordinates)

   Latitude (X, Y°) ______________________________

   Longitude (X, Y°) __________________________

   Altitude (m) ______________________________

   Accuracy (m) ____________________________

3. What is the facility’s catchment population? ____________________
4. What is the facility’s bed capacity? ________________________

B. PATIENTS TRENDS

5. What is the attendance totals for both inpatient and outpatient in the following months

5.1 a) Outpatient for November 2018 ______________

b) Outpatient for December 2018 ______________

c) Outpatient for January 2019_________________

d) Outpatient for February 2019________________

e) Outpatient for March 2019__________________

f) Outpatient for April 2019___________________

g) Outpatient for May 2019____________________

h) Outpatient for November 2019_______________

i) Outpatient for December 2019________________

j) Outpatient for January 2020__________________

k) Outpatient for February 2020 ________________

l) Outpatient for March 2020 __________________

m) Outpatient for April 2020 __________________

n) Outpatient for May 2020____________________

5.2 a) Inpatient for November 2018 ______________

b) Inpatient for December 2018 ______________

c) Inpatient for January 2019__________________

d) Inpatient for February 2019_________________

e) Inpatient for March 2019___________________

f) Inpatient for April 2019____________________

g) Inpatient for May 2019_____________________

h) Inpatient for November 2019________________

i) Inpatient for December 2019_________________

j) Inpatient for January 2020___________________

k) Inpatient for February 2020 _________________

l) Inpatient for March 2020 ___________________

m) Inpatient for April 2020 ___________________

n) Inpatient for May 2020_____________________
